# One-Year Surveillance of SARS-CoV-2 Virus in Natural and Drinking Water

**DOI:** 10.3390/pathogens11101133

**Published:** 2022-09-30

**Authors:** Daniel Salvador, Maria Filomena Caeiro, Célia Neto, Rui Neves Carneiro

**Affiliations:** 1 Direção de Laboratórios (LAB) da Empresa Portuguesa das Águas Livres (EPAL), Avenida de Berlim, 15, 1800-031 Lisboa, Portugal; 2 Centro de Estudos do Ambiente e do Mar (CESAM), Departamento de Biologia Vegetal, Faculdade de Ciências da Universidade de Lisboa, Edifício C2-Piso 4, Campo Grande, 1749-016 Lisboa, Portugal

**Keywords:** human health, *Norovirus*, risk assessment, RT-qPCR, water monitoring, water safety, fecal indicator (FI)

## Abstract

Although the SARS-CoV-2 virus has been detected in wastewater from several countries, monitoring its presence in other water matrices is still limited. This study aimed to evaluate the presence of this virus in natural and drinking water over one year of monitoring (2021). A survey of viral RNA was carried out by RT-qPCR in concentrated samples of surface water, groundwater, and drinking water from different regions of Portugal. SARS-CoV-2 RNA—quantified in genomic copies per liter (gc/L) of sampled water—was not detected in groundwater, but was detected and quantified in samples of surface water (two out of 43; 8035 and 23,757 gc/L) and in drinking water (one out of 43 samples; 7463 gc/L). The study also detected and quantified *Norovirus* RNA, intending to confirm the use of this enteric virus to assess variations in fecal matter throughout the sampling campaign. The samples positive for SARS-CoV-2 RNA also had the highest concentrations of *Norovirus* RNA—including the drinking water sample, which proved negative for fecal enteric bacteria (FIB). These results indicate that, to protect human health, it is advisable to continue monitoring these viruses, and noroviruses as fecal indicators (FI) as well—especially in low-flow water bodies that receive wastewater.

## 1. Introduction

In December 2019, several cases of atypical pneumonia appeared in the Wuhan province in China. After several tests, it was concluded—in January 2020—that they were caused by a new virus from the coronavirus group—SARS-CoV-2 [1,2].

SARS-CoV-2, an enveloped and positive-sense single-stranded RNA virus, is a member of the family *Coronaviridae*, order *Nidovirales* [1,3]. The *Coronaviridae* family includes three subfamilies and the sub-family *Orthocoronavirinae* includes four genera: *Betacoronavirus* (examples: SARS-CoV-2 and MERS-CoV, acronyms of *Severe Acute Respiratory Syndrome-Coronavirus 2* and *Middle East respiratory syndrome-related coronavirus*), *Alphacoronavirus* (example: HCoV-229E, acronym of *Human Coronavirus 229E*), *Gammacoronavirus* (examples are viruses that infect whales and birds), and *Deltacoronavirus* (examples are viruses that infect pigs and birds) [3].

Due to its high transmissibility, SARS-CoV-2 spread around the world and—in March 2021—the resulting disease (COVID-19) was declared by the World Health Organization (WHO) to be a pandemic [2,4]. Worldwide, the current number of confirmed cases is more than 500 million and the number of deaths has already exceeded 6 million [5]. In Portugal, the number of infected is over 5 million inhabitants and the number of deaths was around 24,000 on 5 August 2022 [6].

Given the rapid and continuous transmission of SARS-CoV-2, it is crucial to clearly understand the routes of transmission of this virus to humans so that we can prevent its spread [1,7]. According to current knowledge, its main route of transmission is exposure to droplet sprays or aerosols that can remain for hours in the air [7]. Concerning other routes of transmission, knowledge is still limited, although there is the possibility of transmission by the fecal–oral route. Infected masks released into the environment can also contribute to the contamination of natural water sources [1,4,8]. After infection and multiplication in the body, the most common symptoms are fever, dry cough, fatigue, shortness of breath, and headache [2,9].

SARS-CoV-2 is released by the upper respiratory and gastrointestinal systems. When eliminated through the gastrointestinal system, the excretion through the feces can occur for up to four weeks and wastewater is the main route [2,10]. If this contaminated water is released into the environment without proper treatment, the receiving natural water bodies may also be contaminated, and this could have consequences for human health [1,4].

In recent months, SARS-CoV-2 RNA has been detected in sludge from Wastewater Treatment Plants (WWTPs), municipal wastewater, hospital wastewater, wastewater from cruise ships and commercial passenger aircrafts, non-potable water, treated wastewater (secondary treatment), and surface river water [1,4,8]. Some studies indicate that SARS-CoV-2 can remain infectious for up to several days in wastewater and other coronaviruses can remain viable in these aqueous matrices for more than one year [10].

Most of the studies carried out have focused on wastewater matrices; assessments in other water matrices such as natural or drinking water are limited. In Italy, a study by Rimoldi et al. [11] using detection by RT-PCR found SARS-CoV-2 RNA in water from a river receiving wastewater in April 2020. Haramoto et al. [12] in Japan, between March and May 2020, analyzed wastewater and associated river water and detected and quantified SARS-CoV-2 RNA in treated wastewater (2.4 × 10^3^ gc/L), but not in water from the river. Later, in Mexico, Mahlknecht et al. [13] found SARS-CoV-2 RNA in surface water (3.3–3.8 gc/mL), groundwater (2.6–38.3 gc/mL), river water (2.5–7.0 gc/mL) and wastewater (up to 3535 gc/mL). In Brazil, in a study by Fongaro et al. [14] in August 2020, viral RNA was found in the water of a river downstream of a rural community in Minas Gerais (1.1 × 10^2^ gc/mL). In Nepal, Tandukar et al. [15] detected SARS-CoV-2 RNA in the water of a river at a concentration of 4.0 to 5.0 log10 gc/L, in addition to also having been found in wastewater.

In Portugal, there are to our knowledge no surveillance studies concerning the RNA of SARS-CoV-2 in natural or drinking water. However, several projects directed at wastewater have been developed. In Tomasino et al. [16], SARS-CoV-2 RNA was detected in the solid and liquid phases of untreated wastewater from Porto between May 2020 and March 2021. Monteiro et al. [17] detected and quantified SARS-CoV-2 RNA in wastewater at the entrance of five WWTPs in the metropolitan area of Lisbon and in the north of the country, at concentrations that varied between 10^3^ and 10^5^ gc/L.

In this context, this project intended to carry out a one-year (2021) monitoring of SARS-CoV-2 RNA in natural sources of water and in drinking water distribution systems, through the application of RT-qPCR methodology. The second main objective was the evaluation of the effectiveness of the treatment systems of the WTPs in the elimination of viral RNA. The samples were controlled for fecal contamination via the monitoring of *Norovirus* RNA. The suitability of this virus as a fecal indicator (FI) was under evaluation and should be validated in case of detection of RNA from both viruses (SARS-CoV-2 and *Norovirus*) in samples of water that are negative for the control organisms that are widely, and often exclusively, used for this purpose: fecal indicator bacteria (FIB).

## 2. Materials and Methods

### 2.1. Study Sites and Water Sampling

Natural water sampling included surface water collected at abstraction sites located at a river and four dam/reservoirs and three groundwater sources/boreholes. All water bodies are currently used to produce drinking water that is supplied to various municipalities of the country. The characterization of the sampling sites is shown in Table 1. The areas around the various bodies of water include large agricultural fields, animal production units, and industries.

The sampling campaigns were carried out between January and December 2021. Sampling of surface water was carried out at the water inlet of the WTPs, before any treatment. After treatment, sampling of drinking water was carried out at the outlet of the WTPs. Sampling of untreated groundwater was performed at the outlet of the boreholes. After a disinfection treatment step, the respective treated groundwater was collected—just before entering the distribution network.

The collection procedures were performed as described in Salvador et al. [18], with adjustments in the filtered volumes (Salvador et al. [19]). High volumes (70 to 250 L) of water were collected at the sampling points and concentrated by filtration with NanoCeram filters (Argonite; Sanford, FL, USA). After filtration, carried out in field, the filter with sample was transported under refrigeration to the laboratory. Sample processing was performed up to 72 h after sampling.

### 2.2. Processing and Concentration of the Filtered Water Samples

The processing of the samples was carried out according to Salvador et al. [18], also taking into account the results described in Salvador et al. [19]. In summary, in the laboratory, the NanoCeram filters (Argonite; Sanford, FL, USA) with the samples were eluted with 3% beef extract (BD Bios-science; Franklin Lakes, NJ, USA), followed by an organic flocculation process (45 min) and several steps of concentration by centrifugation, resuspension of the sediment with sodium phosphate pH 7.0–7.5, and a final filtration through 0.22 µm pore-size Acrodisc Syringe filters (PALL Corporation; Ann Arbor, MI, USA). The resulting volume (about 32 mL) was aliquoted and kept at -70 °C until the next step. Of the 32 mL, about 20 mL were used for RNA extraction and 12 mL for storage. In the secondary concentration step, the samples were applied in Vivaspin concentrators (Sartorius; Goettingen, Germany) and centrifuged at 8000× *g* and 4 °C for 6 h. Finally, the concentrates were subjected to RNA extraction and purification with the viral QIAamp RNA Mini kit (Qiagen; Hilden, Germany), according to the manufacturer’s instructions.

### 2.3. Detection and Quantification of SARS-CoV-2 RNA

RT-qPCR reactions for the detection and quantification of SARS-CoV-2 genomic RNA were performed on a StepOnePlus thermocycler (Applied Biosystems; Foster City, CA, USA). The viral RNA was assayed with a SARS-CoV-2 RT-PCR Test (IDEXX Laboratories; Westbrook, Maine, USA) in reaction mixtures of 20 µL containing 5 µL of extracted RNA. The primers, described in Table 2, targeted the N gene (N1 and N2 regions). The amplification conditions were the following: reverse transcription at 50 °C for 15 min, denaturation at 95 °C for 1 min followed by 45 cycles of amplification at 95 °C for 15 s, and data collection at 60 °C for 30 s. Quantification of SARS-CoV-2 was estimated by standard curves, with five points constructed with serial dilutions (3000 µg/µL; 1500 µg/µL; 150 µg/µL; 15 µg/µL; and 2 µg/µL) of a positive control (SARS-CoV-2 RNA at 15,000 µg/µL; Vircell; Granada, Spain). In the RT-qPCR runs, each sample was tested in duplicate. Positive and negative controls were added. The results are the average value of two independent amplifications for each sample. They are expressed in genomic copies per liter of collected sample (gc/L). Only samples with Ct (cycle threshold) values below 40 were considered positive. Only results that satisfied the quality requirements specified by the kit instructions were considered. Data processing was performed using Microsoft Excel 2017 (Microsoft Inc., Redmond, WS, USA).

### 2.4. Detection and Quantification of Norovirus RNA

To evaluate the possible presence of fecal matter in samples of natural and drinking water and its variation over time, a ubiquitous enteric virus was monitored: *Norovirus*. Note that current methods for the detection, identification, and quantification of FIB were routinely applied. The procedures for the FIB evaluation, as well as the procedures for the detection and quantification of *Norovirus* RNA, were performed in accordance with Salvador et al. [21]. For norovirus RNA, the amplifications were performed in a StepOnePlus thermocycler (Applied Biosystems; Foster City, CA, USA); *Norovirus* Genogrup I was assayed with a NorovirusGI Kit (bioMérieux; Marcy-l’Etoile, France), and *Norovirus* Genogrup II with a CeeramTools NorovirusGII Kit (bioMérieux; Marcy-l’Etoile, France). The reaction volume was 25 µL, including 5 µL of extracted RNA, and the amplification conditions were the same for both: reverse transcription at 45 °C for 10 min, polymerase enzyme activation at 95 °C for 10 min followed by 45 amplification cycles with denaturation at 95 °C for 15 s, and data collection at 60 °C for 45 s. Each sample was evaluated in duplicate. The quantification of *Norovirus* was performed using standard curves with five points, constructed with serial dilutions (1:10) of control RNA of NoV I or NoV II (CeeramTools NorovirusGI Standard kit; bioMérieux; Marcy-l’Etoile, France) with a starting concentration of 2.0 × 10^6^ gc/µL. Only samples with a Ct below 40 were considered positive. The processing of the results was performed using Microsoft Excel 2017 (Microsoft Inc., Redmond, WS, USA).

## 3. Results

### 3.1. Monitoring of SARS-CoV-2 RNA in Natural and Drinking Water

**Figure 1:**
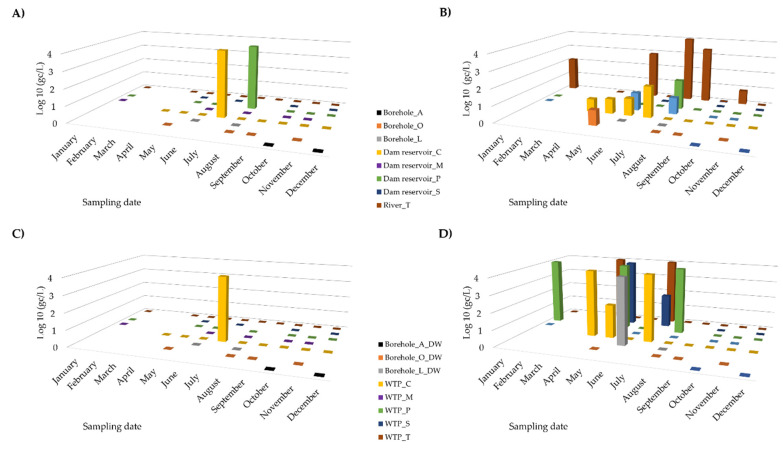
Variation in the concentrations of SARS-CoV-2 and *Norovirus* RNAs throughout the 2021 sampling campaign in sources of natural water (*n* = 43) and drinking water (*n* = 43). (**A**) SARS-CoV-2 RNA concentration in natural water. (**B**) *Norovirus* RNA concentration in natural water. (**C**) SARS-CoV-2 RNA concentration in drinking water. (**D**) *Norovirus* RNA concentration in drinking water. Each concentration value, in Log10 (gc/L), is the average of two independent RT-qPCR results.

Between January and December 2021, 43 samples of natural water were collected—35 came from surface water bodies and eight from boreholes. SARS-CoV-2 RNA was only detected in two surface water samples—one from Dam reservoir_C and another from Dam reservoir_P (Table 3). The positive sample from Dam reservoir_C was collected in July and had a concentration of 23,757 gc/L and the positive sample of Dam reservoir_P, with a viral RNA concentration of 8035 gc/L, was collected in August (Table 3, Figure 1A).

During 2021, 43 samples of drinking water were collected at the outlet of WTPs or groundwater disinfection plants. SARS-CoV-2 RNA was only detected in a water sample from WTP_C in July, at a concentration of 7463 gc/L (Table 3, Figure 1C).

### 3.2. Evaluation of the Electiveness of Water Treatments in the Elimination of SARS-CoV-2 RNA

SARS-CoV-2 RNA was detected in July, both in the natural surface water from Dam reservoir_C and in the drinking water collected at the outlet of WTP_C. A reduction of 69% (23,757 gc/L to 7463 gc/L) was observed after the treatment process (Figure 1A,C).

The SARS-CoV-2 RNA detected in August in the natural surface water from Dam reservoir_P suffered a total reduction (100%) as it was not detected in drinking water at the outlet of the WTP_P (Figure 1A,C).

In the remaining samples, SARS-CoV-2 RNA was not found in either sources of natural water nor in the corresponding treated drinking water.

## 4. Discussion

SARS-CoV-2 RNA was detected in two natural surface water sources (Dam reservoir_C and Dam reservoir_P), as had already happened in other countries—namely, Italy [11], Mexico [13], Brazil [14], and Nepal [15]. However, in the referred studies, the viral RNA was detected in water samples taken from rivers—not from dam reservoirs, as occurred in the present project. Most of the work on the environmental monitoring of SARS-CoV-2 and other viruses focuses on wastewater matrices, with little knowledge of natural or drinking waters matrices, whose monitoring is extremely important because many of these water bodies are associated with the supply of water to populations and their activities [22,23].

SARS-CoV-2 RNA was detected and quantified in two of the 43 natural water samples, with concentrations of 23,757 gc/L and 8035 gc/L—higher than those found by Mahlknecht et al. [13] in surface water (3.3–3.8 gc/mL) and in river waters (2.5–7.0 gc/mL) from Mexico, and lower than the concentrations found in the study by Fongaro et al. [14] in river water downstream of a rural community of Brazil (1.1 × 10^2^ gc/mL), or in the work of Tandukar et al. [15] in the water of a river from Nepal (4.0 to 5.0 log10 gc/L).

The presence of SARS-CoV-2 RNA in surface water may be associated with illicit discharges of untreated sewage with feces from infected individuals, discharges of improperly treated wastewater, the malfunction of sewer systems, and discharges of wastewater from hospitals [7,11].

SARS-CoV-2 RNA-positive samples were collected during the summer months in Portugal (July and August), when—associated with the high prevalence of infections in the population—precipitation is rare and high temperatures lead to greater evaporation, thus resulting in a decrease in river flows as well as in the water levels in dam reservoirs [7,11]. Under these conditions, discharges of contaminated water into the natural environment have a greater impact—revealed, for example, in the detection of viral RNA, which may be the explanation for the higher viral RNA concentrations of *Norovirus* (enteric virus) found during these months. It is also important to highlight the risk of contamination via the ingestion of food prepared with vegetables irrigated with these contaminated waters [23].

The available knowledge regarding the occurrence of viruses in groundwater is still scarce and much remains to be clarified. In this study, all the samples collected from the three boreholes were negative for SARS-CoV-2—but in Mahlknecht et al. [13], viral RNA was detected and quantified in groundwater from Mexico at a concentration of 2.6–38.3 gc/mL. According to Wyk et al. [24], contamination of this matrix with SARS-CoV-2 is unlikely due to the physical protection of these water bodies (proportional to the depth where they are located).

In drinking water, SARS-CoV-2 RNA was detected in one sample from WTP_C. For this type of matrix, there are no known studies carried out on the monitoring of this virus—although several environmental management and public health institutions, such as the U.S. Environmental Protection Agency, recommend its monitoring for safety reasons [25,26]. Realizing that viral RNA was only detected in one sample from the 43 analyzed, and considering the properties of the virus (to be mentioned below), it is very unlikely it is transmitted to humans via drinking water.

The efficiency of the water treatment methods, regarding the removal of SARS-CoV-2 RNA, was different in the two WTPs that treated the contaminated natural surface waters. In the WTP_P, the viral RNA seemed to have been eliminated—considering that it was not detected in the treated drinking water outlet. In WTP_C, the viral RNA was only reduced by a yield of 69%. This finding may be associated with the fact that, unlike WTP_C, WTP_P has a pre-oxidation step with ozone at the beginning of the treatment process, and ozone has been pointed out as having high potential in the control and elimination of viruses [21,27]. On the other hand, enteric viruses are much more resistant to water treatments [28], as could be seen in the present study, where *Norovirus* RNA was detected in a large number (11) of drinking water samples—WTP_P samples included. This emphasizes that the water from these WTPs was, as usual, negative for FIB [21].

The RT-PCR method—fundamental in the monitoring of pathogens and in the management of outbreaks caused by contact with contaminated water—has a high specificity and sensitivity but only allows the detection and quantification of viral RNA present in the sample, not evaluating its integrity and—even less so—its viral infectivity [29,30,31].

Although no analyses were carried out on the infectivity of the three samples where SARS-CoV-2 RNA was detected, it is expected that they only consisted of RNA—possibly not even intact and very likely without infectivity, as noticed in Rimoldi et al. [11]—these authors detected SARS-CoV-2 RNA both in river water and wastewater; however, without finding the samples developing cytopathic effects (CPE) in infectivity tests. Despite the fact that coronaviruses can persist for up to several weeks in aquatic environments [30], this lack of infectivity is expected, considering that their enveloped virions are more susceptible to deterioration and loss of infectivity than the naked virions of many enteroviruses (e.g., noroviruses) [4]. The structure of SARS-CoV-2 is strongly affected by the properties of water, such as temperature, pH, concentration of suspended solids, concentration of organic matter, and dose and type of disinfectants used [4,31]. In contrast, non-enveloped enteric viruses are much more resistant to environmental conditions—particularly when associated with organic matter [4,29].

To monitor the presence of fecal matter in the water samples, the concentration of *Norovirus* RNA was evaluated. *Norovirus* is an enteric virus that replicates in the gastrointestinal tract, and—like SARS-CoV-2—it is also excreted in the feces of infected individuals several weeks after infection [4,29]. They are non-enveloped viruses belonging to *Caliciviridae* and are classified into six genogroups (GI to GVI)—of which, only representatives of the genogroups I, II, and IV infect humans [32]. *Norovirus* RNA (considering together the genotypes I and II, evaluated in the present survey) was detected in just one sample of groundwater, 12 samples of surface water, and 11 samples of drinking water. These results clearly indicate the contamination of surface water with fecal matter, where noroviruses are commonly detected—also evidencing the low efficacy of the WTPs in removing viral RNA, further detected in the treated drinking water. This was as previously reported in Salvador et al. [18] and Salvador et al. [21], in surveys also targeting other enteric viruses where FIB was not detected in drinking water—as in the present study.

Considering the fecal transmission route of SARS-CoV-2, it is likely that the samples positive for SARS-CoV-2 RNA also evidenced the presence of *Norovirus* RNA, due to the association of both viruses with fecal matter from populations sporadically affected by COVID-19, whose droppings reached the sampled water. This finding once more confirmed that *Norovirus*, like other enteric viruses, is a good indicator of fecal contamination. Therefore, the presence/absence of noroviruses in a particular water source allows one to anticipate its vulnerability to fecal contamination and to propose more adequate surveillance measures [21]. These results reinforce previous studies [21] proposing that, instead of FIB, enteric viruses (e.g., noroviruses) should be considered adequate FI in the monitoring of viruses (SARS-CoV-2 included) in drinking water.

Despite the above considerations concerning SARS-CoV-2 viability in the environment, in agreement with Mancuso et al. [23] and considering the results of this study, we recommend future studies focused on the assessment of the infectivity of SARS-CoV-2 in different water matrices and in different environmental scenarios whenever viral RNA is detected.

## 5. Conclusions

SARS-CoV-2 RNA was detected and quantified for the first time in Portugal in natural surface water and drinking water. In natural groundwater, it was not found.

The RNA of this virus was found in a small number of samples, and the absence of associated viral infectivity is anticipated considering its enveloped nature, which confers high sensitivity to environmental conditions and water treatments.

The monitoring of enteric virus—*Norovirus*—allowed us to evaluate the presence of fecal matter in the samples throughout the sampling campaign and to estimate the level of contamination of natural waters with wastewater. The detection of noroviruses in drinking water negative for FIB reinforces previous recommendations for using enteric viruses as FI in the surveillance of viruses.

Taking these results into account and to protect human health, it is recommended to continue monitoring these and other potentially pathogenic viruses—especially in water bodies that receive wastewater or present with low flows.

## Figures and Tables

**Table 1:** Characterization of the natural and drinking water sampling sites.

Water Matrix	Water Source	Water Treatment Plant	Region *	Treatment Schemes	Observations
Surface water	River	WTP_T	Lisboa e Vale do Tejo	Pre-oxidation with potassium permanganate and/or ozone, pH adjustment, coagulation-flocculation, adsorption, decantation, oxidation with sodium hypochlorite, filtration, pH correction, and final disinfection	Water Treatment Plant composed of two independent treatment lines, each with the capacity to produce 120,000 m^3^/day
	Dam reservoir_C	WTP_C	Lisboa e Vale do Tejo	Pre-chlorination, aggressiveness correction and remineralization, coagulation-flocculation, flotation, filtration, pH adjustment, and final disinfection	Water Treatment Plant composed of two independent treatment lines, with the capacity to produce 500,000 m^3^/day (line 1) and 125,000 m^3^/day (line 2)
	Dam reservoir_M	WTP_M	Alentejo	Ozone pre-oxidation, remineralization, coagulation, addition of activated carbon, flocculation-flotation, flocculation-decantation, manganese removal, filtration, pH adjustment, and final disinfection	Water Treatment Plant with the capacity to produce 26,400 m^3^/day
	Dam reservoir_P	WTP_P	Alentejo	Chemical pre-oxidation with ozone, pH correction, remineralization, coagulation, flocculation, flotation, intermediate oxidation, filtration, pH correction, and final disinfection	Water Treatment Plant with the capacity to produce around 16,800 m^3^/day
	Dam reservoir_S	WTP_S	Centro	Pre-oxidation with ozone, remineralization, coagulation/flocculation, adsorption with activated carbon, decantation, filtration, pH adjustment, and disinfection	Water Treatment Plant with the capacity to produce around 2200 m^3^/hour
Groundwater	Borehole_A	-	Lisboa e Vale do Tejo	Disinfection with sodium hypochlorite	Disinfection is carried out by a system with hypochlorite at the exit of the water from the borehole and before entering the network. There are no associated WTPs
	Borehole_L	-			
	Borehole_O	-			

* Territorial distribution according to the Comissão de Coordenação e Desenvolvimento Regional de Portugal (https://www.ccdr-lvt.pt/, accessed on 30 August 2022).

**Table 2:** Primers and probes of RT-qPCR assays used in this study.

Name	N Gene Region	Function	Sequence (5′–3′)	Reference
**2019-nCoV_N1-F**	**N1**	**Forward Primer**	GACCCCAAAATCAGCGAAAT	Centers for Disease Control and Prevention [20]
**2019-nCoV_N1-R**		**Reverse Primer**	TCTGGTTACTGCCAGTTGAATCTG	
**2019-nCoV_N1-P**		**Probe**	FAM-ACCCCGCATTACGTTTGGTGGACC-BHQ1	
**2019-nCoV_N2-F**	**N2**	**Forward Primer**	TTACAAACATTGGCCGCAAA	
**2019-nCoV_N2-R**		**Reverse Primer**	GCGCGACATTCCGAAGAA	
**2019-nCoV_N2-P**		**Probe**	FAM-ACAATTTGCCCCCAGCGCTTCAG-BHQ1	

**Table 3:** Detection and quantification of SARS-CoV-2 and *Norovirus* RNA in natural (*n* = 43) and drinking water (*n* = 43) during 2021.

Water Matrix		Water Source	Number of Collected Samples	SARS-CoV-2 RNA		*Norovirus* RNA Genogroup I + Genogroup II	
				Number of Positive Samples	Average Concentration (gc/L) *	Number of Positive Samples	Average Concentration (gc/L) *
**Natural water**	**Surface water**	**River_T**	10	0	-	5	4132
		**Dam reservoir_C**	9	1	23,757	4	26
		**Dam reservoir_M**	5	0	-	2	11
		**Dam reservoir_P**	7	1	8035	1	60
		**Dam reservoir_S**	4	0	-	0	-
	**Groundwater**	**Borehole_A**	2	0	-	0	-
		**Borehole_L**	2	0	-	0	-
		**Borehole_O**	4	0	-	1	8
**Drinking water from surface water**		**WTP_T**	10	0	-	2	263
		**WTP_C**	9	1	7463	3	188
		**WTP_M**	5	0	-	0	-
		**WTP_P**	7	0	-	3	16
		**WTP_S**	4	0	-	2	7
**Drinking water from groundwater**		**Borehole_A_DW**	2	0	-	0	-
		**Borehole_L_DW**	2	0	-	1	18
		**Borehole_O_DW**	4	0	-	0	-

* Mean of duplicate RT-qPCR analyses.
